# Use of proton pump inhibitors to treat persistent throat symptoms: multicentre, double blind, randomised, placebo controlled trial

**DOI:** 10.1136/bmj.m4903

**Published:** 2021-01-07

**Authors:** James O’Hara, Deborah D Stocken, Gillian C Watson, Tony Fouweather, Julian McGlashan, Kenneth MacKenzie, Paul Carding, Yakubu Karagama, Ruth Wood, Janet A Wilson

**Affiliations:** 1 Freeman Hospital, Newcastle upon Tyne Hospitals NHS Foundation Trust, Newcastle upon Tyne NE7 7DN, UK; 2 Population Health Sciences Institute, Newcastle University, Newcastle upon Tyne, UK; 3 Clinical Trials Research, Leeds Institute of Clinical Trials Research University of Leeds, Leeds, UK; 4 Newcastle Clinical Trials Unit, Newcastle University, Newcastle, UK; 5 Population Health Sciences Institute, Newcastle University, Newcastle upon Tyne, UK; 6 Nottingham University Hospitals, Nottingham, UK; 7 NHS Greater Glasgow and Clyde. Visiting Professor, University of Strathclyde, Glasgow, UK; 8 Oxford Institute of Nursing, Midwifery and Allied Health Research, Oxford Brookes University, Oxford, UK; 9 Guy’s and St Thomas’ NHS Foundation Trust, London, UK; 10 Population Health Sciences Institute, Newcastle University and Newcastle upon Tyne Hospitals NHS Foundation Trust, Newcastle upon Tyne, UK

## Abstract

**Objective:**

To assess the use of proton pump inhibitors (PPIs) to treat persistent throat symptoms.

**Design:**

Pragmatic, double blind, placebo controlled, randomised trial.

**Setting:**

Eight ear, nose, and throat outpatient clinics, United Kingdom.

**Participants:**

346 patients aged 18 years or older with persistent throat symptoms who were randomised according to recruiting centre and baseline severity of symptoms (mild or severe): 172 to lansoprazole and 174 to placebo.

**Intervention:**

Random blinded allocation (1:1) to either 30 mg lansoprazole twice daily or matched placebo twice daily for 16 weeks.

**Main outcome measures:**

Primary outcome was symptomatic response at 16 weeks measured using the total reflux symptom index (RSI) score. Secondary outcomes included symptom response at 12 months, quality of life, and throat appearances.

**Results:**

Of 1427 patients initially screened for eligibility, 346 were recruited. The mean age of the study sample was 52.2 (SD 13.7) years, 196 (57%) were women, and 162 (47%) had severe symptoms at presentation; these characteristics were balanced across treatment arms. The primary analysis was performed on 220 patients who completed the primary outcome measure within a window of 14-20 weeks. Mean RSI scores were similar between treatment arms at baseline: lansoprazole 22.0 (95% confidence interval 20.4 to 23.6) and placebo 21.7 (20.5 to 23.0). Improvements (reduction in RSI score) were observed in both groups—score at 16 weeks: lansoprazole 17.4 (15.5 to19.4) and placebo 15.6 (13.8 to 17.3). No statistically significant difference was found between the treatment arms: estimated difference 1.9 points (95% confidence interval −0.3 to 4.2 points; P=0.096) adjusted for site and baseline symptom severity. Lansoprazole showed no benefits over placebo for any secondary outcome measure, including RSI scores at 12 months: lansoprazole 16.0 (13.6 to 18.4) and placebo 13.6 (11.7 to 15.5): estimated difference 2.4 points (−0.6 to 5.4 points).

**Conclusions:**

No evidence was found of benefit from PPI treatment in patients with persistent throat symptoms. RSI scores were similar between the lansoprazole and placebo groups after 16 weeks of treatment and at the 12 month follow-up.

**Trial registration:**

ISRCTN Registry ISRCTN38578686 and EudraCT 2013-004249-17.

## Introduction

Persistent throat symptoms are a common presentation in primary and secondary care and principally comprise hoarseness; the sensation of a lump in the throat (globus); repeated throat clearing; mucus in the throat, or “catarrh”; cough; and throat discomfort. The prevalence of globus alone in middle aged women is about 6%,^1^ with a lifetime ever population incidence of more than 40%.^2^ A quarter of patients attending primary care for other conditions might report major throat symptoms when questioned.^3^

Gastroesophageal reflux disease (GORD) affects up to 20% of the Western population.^4^ An association between GORD and throat and voice symptoms is widely cited—a decade ago more than half of British otolaryngologists prescribed proton pump inhibitors (PPIs) for throat symptoms.^5^ GORD and related symptoms have been described using a variety of terms, including extraoesophageal reflux, laryngopharyngeal reflux, and reflux laryngitis. The concept of a link between GORD and throat and voice symptoms has since become even more popular, with open access primary care guidelines advocating PPI treatment.^6^ The few randomised controlled trials that have compared PPIs with placebo are heterogeneous and generally underpowered.^7^ By far the largest controlled trial included 145 patients^8^ and found no benefit from treating suspected reflux laryngitis with PPIs twice daily compared with placebo twice daily. Despite variable quality, recent meta-analyses of small randomised controlled trials indicate either no benefit^9^ or mild superiority^10^ ^11^ of PPIs over placebo. Increasing use of PPIs, at cost to the National Health Service, has become the default treatment for persistent throat symptoms in primary and secondary care without robust evidence. Inappropriate use of PPIs is a major healthcare concern and contributes to polypharmacy, risk of drug interactions, and risk of side effects. A US study found that the cost of treating extraoesophageal reflux symptoms in 281 patients was more than fivefold that of the estimated cost of treating patients with traditional GORD symptoms,^12^ with more than 50% of these costs attributed to prescriptions for PPIs.

We investigated the role of PPIs as pragmatic preferred treatment for throat symptoms in primary and secondary care.

## Methods

This trial was an investigator initiated, multicentre, randomised, double blind, placebo controlled trial conducted in eight hospitals in the UK. The trial protocol was approved by the regional ethics committee and has been published previously.^13^ Both an independent data monitoring committee and a trial steering committee oversaw the trial.

### Patients and presenting characteristics

Participants were adults (≥18 years) newly referred to eight secondary care otolaryngology clinics with persistent (>6 weeks) unexplained throat symptoms—principally hoarseness, throat pain, globus sensation, throat clearing, postnasal secretions or excess mucus, cough, or choking sensation. Given the prevalence of minor throat symptoms in the general population, we considered patients to be eligible for the trial based on symptom severity. At baseline we assessed severity using the reflux symptom index (RSI), a well established patient self-report questionnaire (see supplementary table 1),^14^ which is widely used in voice and general otolaryngology clinics. It is also one of the few tools with published data on sensitivity to change^15^ and with normative ranges for the general population.^16^ The last of the nine items of the RSI is a composite GORD question covering heartburn, chest pain, indigestion, or stomach acid reflux. Although the upper limit of the RSI population norm is generally taken to be 12 points, at least 5 of the 12 points can be achieved by maximum endorsement of dyspepsia (item 9). To ensure that patients had a qualifying level of severity for the non-dyspepsia items (ie, the throat symptoms in question), all participants were required to score at least 10 points on items 1 to 8 of the RSI (here referred to as RSI-HB to denote the laryngopharyngeal RSI items without the heartburn score). We excluded patients if laryngopharyngeal endoscopy showed disease requiring specific treatment, such as vocal cord polyps or malignancy, or they had a contraindication to PPIs. Patients currently taking a PPI required a wash-out period of four weeks to enter the trial, and those taking alginates were required to discontinue these drugs. The complete list of inclusion and exclusion criteria are published elsewhere.^13^ To ensure consistency across the sites, all participants had access to the trial website and introductory video before providing written informed consent.

### Trial procedures

The active intervention was 30 mg lansoprazole twice daily for 16 weeks. The control group received matched placebo capsules twice daily for 16 weeks. Lansoprazole was chosen as a representative PPI because of its popularity and continued inclusion in UK national guidance for the treatment of GORD, in which the dosage regimen equates to a high or double dose.^17^ No evidence supports the superiority of one PPI over another for persistent throat symptoms. The participants and research team staff were blinded to treatment allocation, which was maintained throughout the trial. Participants were assessed at baseline (after informed consent) and at 16 weeks and 12 months. After the assessment at 16 weeks, they were not prescribed any trial drug for symptoms. Randomisation was administered centrally through the Newcastle Clinical Trials Unit using a secure web based system. Patients were randomised using permuted random blocks, and allocated 1:1 stratified by centre and baseline symptom severity on the basis of a dichotomised RSI-HB score (mild ≤20, severe >20) to ensure the population reflected persistent throat symptoms and not classic GORD symptoms. We used severity stratums derived from data in published RSI datasets.^15^ ^18^

### Outcome measures

The primary outcome measure was the total RSI score (a summation of all nine items) at 16 weeks after randomisation. The RSI was scored on a 6 point Likert scale (0-5), giving a total range of 0-45 scores (see supplementary table 1). A higher score indicates more severe symptoms.

The secondary outcomes were compliance with the intervention, as measured by reported drugs taken and return of unused tablets (treatment kits contained a 16 week course of 224 capsules), RSI score at 12 months after randomisation, two further patient self-report symptom measures—the 34 item comprehensive reflux symptom score (CReSS)^19^ and the 43 item laryngopharyngeal health related quality of life tool^20^ (higher scores for both equate to more severe symptoms), and utility of baseline laryngeal mucosal changes (all participants were assessed by a single clinician, who was blind to the symptom scores) recorded by the reflux finding score^21^ as a predictor of outcome.

These secondary outcomes were prespecified in the published protocol^13^ and defined in the ISRCTN registry. The main trial outcomes (primary outcome measure, secondary outcome of RSI score at 12 months, and adverse events) are reported in EudraCT. Several secondary outcomes were not defined in the trial registry owing to an error by the authors and therefore must be considered as post hoc additions. These were total laryngopharyngeal item RSI score (omitting the GORD item, RSI-HB, score 0-40), patient post-treatment prediction of allocated intervention, and patient satisfaction with the trial. Other than the participant post-treatment prediction of allocated intervention secondary outcome, the other outcomes were defined in the published protocol.

### Statistical analysis

For the main analysis of the primary outcome measure we used a multivariable multilevel mixed effect linear regression to compare the RSI at 16 weeks after adjustment for the stratification factors at randomisation, with centre as a random effect and mild or severe baseline severity categories as a fixed effect. As the trial proceeded it became clear to the trial steering committee that some participants had considerably delayed their primary outcome and data collection follow-up appointments. The trial steering committee recommended that the primary analyses be based on a compliant intention-to-treat group because of concerns that responses beyond 20 weeks would not be representative of the time impacted by the 16 week course of treatment. This amendment to the published trial protocol was approved within the statistical analysis plan. The primary intention-to-treat analysis was therefore performed on a compliant group of patients (those who completed the 16 week primary outcome within a 14-20 week window), retaining patients in their randomised group. Secondary intention-to-treat analyses were performed on the pragmatic group—that is, all participants who completed the primary outcome, to include those additional patients seen after 20 weeks for their primary outcome assessment. Secondary analyses of the primary outcome also included adjustment for reflux finding score as a continuous measure (investigating non-linear relations using first order fractional polynomial transformation) and for other important clinical and personal baseline factors.

Analyses of secondary outcomes followed a similar strategy for questionnaire scores. We did not compare safety data statistically. Other data were analysed using statistical software package (STATA14). A statistical analysis plan following published guidance^22^ was in place before comparative analyses. No formal interim analyses were planned.

We aimed to recruit 332 patients. A mean difference of 3 points in RSI score at 16 weeks was deemed to be clinically important. With an assumed standard deviation of 7.7^23^ a mean difference of 3.1 points equates to a standardised mean effect size of 0.4. A total of 266 participants (133 in each arm) were required to complete the trial intervention to be able to detect this standardised effect size with 90% power and 5% significance, inflated to 332 participants (166 in each arm) to allow for 20% drop-out.

### Patient and public involvement

Patients were involved in the design of the trial at the grant application stage and attended meetings of the trial steering committee. They helped define the need to explore the clinical management of throat and voice symptoms and aided the research team with the methodology, in particular confirming the appropriateness of the selected patient reported outcome measures. They will not be informed individually of the trial results, but the findings will be openly available on the trial’s website (www.toppits.co.uk) after publication.

## Results

**Fig 1:**
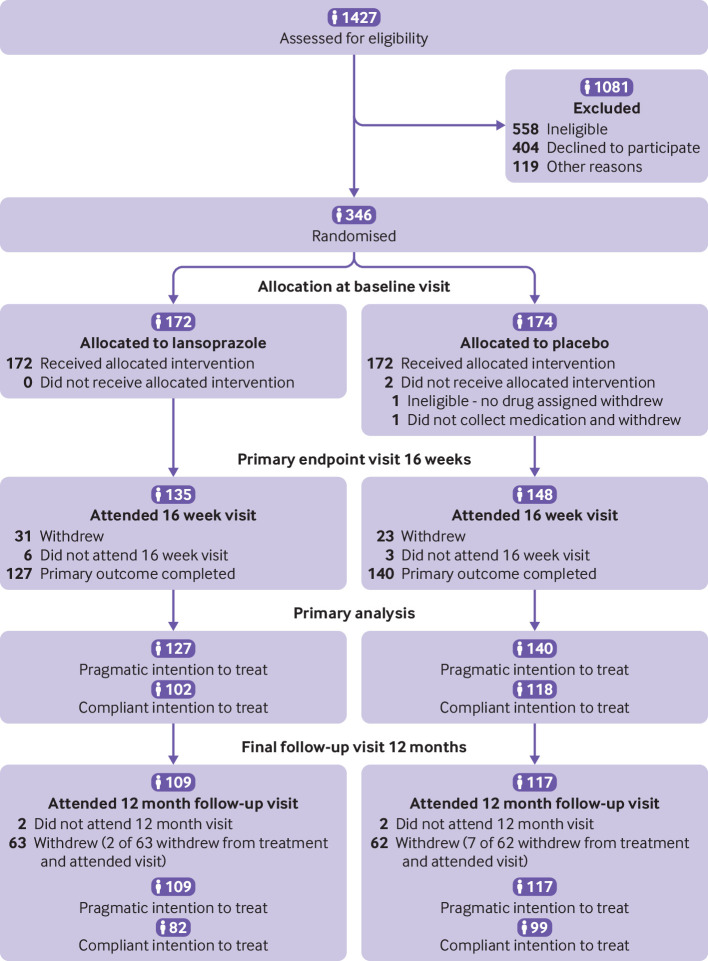
Flow of participants through study

Of 1427 patients initially screened for eligibility, 346 were recruited through eight UK centres and randomised between April 2014 and February 2017: 172 allocated to lansoprazole and 174 to placebo (fig 1). Seventy (27%) of the recruited participants had received PPIs in the previous 12 months, and this was balanced across treatment groups. The drop-out rate was as anticipated in the design and was not different across treatment groups. Overall, 267 (77%) participants completed the 16 week primary outcome measure (the pragmatic intention-to-treat group), 220 of whom completed it within the specified 14-20 week window (the compliant intention-to-treat group). RSI questionnaires returned at 16 weeks were fully completed.

The compliant intention-to-treat group was representative of the trial population for personal characteristics (table 1): 126 (57%) were women, mean age was 54.5 (SD 13.1) years, and 107 (49%) had severe RSI-HB and overall mean RSI-HB scores of 20.0 (SD 7.0) points, balanced across treatment arms (see supplementary table 2 for details of personal characteristics).

**Table 1:** Baseline characteristics of participants

Variables	Participants				Compliant ITT group*		
	Lansoprazole (n=172)	Placebo (n=174)	Total (n=346)		Lansoprazole (n=102)	Placebo (n=118)	Total (n=220)
No (%) men	71 (41)	79 (45)	150 (43)		38 (37)	56 (47)	94 (43)
No (%) women	101 (59)	95 (55)	196 (57)		64 (63)	62 (53)	126 (57)
Age (years):							
Mean (SD)	53.5 (13.3)	50.8 (13.9)	52.2 (13.7)		55.3 (12.8)	53.8 (13.4)	54.5 (13.1)
Range	21-84	20-80	20-84		23-84	21-80	21-84
Body mass index:							
Mean (SD)	28.2 (5.9)	28.1 (5.3)	28.1 (5.6)		28.5 (6.7)	28.4 (5.4)	28.5 (6.1)
Range	11.3-56.9	18.3-49.1	11.3-56.9		11.3-56.9	18.3-49.1	11.3-56.9
Baseline RSI score:							
Mean (SD)	21.7 (7.4)	22.1 (7.0)	21.9 (7.2)		22.0 (8.0)	21.7 (7.1)	21.9 (7.5)
Range	10-41	10-43	10-43		10-41	10-43	10-43
Baseline severity RSI-HB:							
Mean (SD)	20.0 (6.8)	20.1 (6.5)	20.1 (6.6)		20.3 (7.4)	19.8 (6.6)	20.0 (7.0)
Range	10-38	10-38	10-38		10-38	10-38	10-38
Severity category†:							
Mild	91 (53)	93 (53)	184 (53)		53 (52)	60 (51)	113 (51)
Severe	81 (47)	81 (47)	162 (47)		49 (48)	58 (49)	107 (49)

ITT=intention to treat; RSI=reflux symptom index; RSI-HB=laryngopharyngeal RSI items without the heartburn score.

* Only includes patients who completed the 16 week primary outcome measure within the 14 to 20 week window.

† Stratification factor at randomisation: mild (10 ≤RSI-HB ≤20), severe (RSI-HB >20).

### Treatment

In total, 265 of 346 (77%) participants had information on returned trial drug, of whom 262 (99%) were reported to have started treatment, taking at least one capsule. Of these 262 participants, 184 (70%) reported taking at least 90% of the full dose, balanced across treatment groups.

In total, 112 adverse events were reported in 74 patients, 80 (71%) of which occurred during treatment: 42 (70%) in the lansoprazole group and 38 (73%) in the placebo group. One severe adverse event, a rash that appeared after taking the allocated treatment, was categorised as probably related to treatment.

When participants were asked post-treatment to predict their allocated intervention, 42% of the lansoprazole group and 56% of the placebo group correctly identified their treatment at the end of the trial period. At 12 months, of the 213 responders, 54% were very satisfied and 28% were satisfied with the trial.

### Primary outcome measure

An improvement in RSI (reduction in score) was observed overall in the compliant intention-to-treat group at 16 weeks, with a reduction in mean score from 21.9 (SD 7.5) at baseline to 16.4 (SD 9.9). This improvement was observed in both treatment groups (table 2). Multilevel mixed effect linear regression of the RSI score at 16 weeks, adjusted for stratification factors at randomisation (site and RSI-HB severity category) showed baseline RSI-HB to be statistically significantly related to the RSI score at 16 weeks (table 3). The RSI score at 16 weeks was estimated to be 8 points higher (worse) in participants in the severe severity stratum at baseline than those in the mild symptom severity stratum. No statistically significant difference was found in RSI scores at 16 weeks between treatment groups: after adjustment for stratification factors, the lansoprazole group scored 1.9 points higher (worse) than the placebo group (95% confidence interval −0.3 to 4.2, P=0.096). Supplementary table 3 displays the individual RSI items scores at baseline and 16 weeks.

**Table 2:** Questionnaire outcome scores for compliant intention-to-treat group

Questionnaires and intervention	No in group	Mean score at follow-up (95% CI)		
		Baseline	16 weeks*	12 months
RSI*:				
Lansoprazole	102	22.0 (20.4 to 23.6)	17.4 (15.5 to 19.4)	16.0 (13.6 to 18.4)
Placebo	118	21.7 (20.5 to 23.0)	15.6 (13.8 to 17.3)	13.6 (11.7 to 15.5)
Difference†		0.3 (−1.7 to 2.3)	1.8 (−0.8 to 4.4)	2.4 (−0.6 to 5.4)
RSI-HB:				
Lansoprazole	102	20.3 (18.8 to 21.7)	16.3 (14.5 to 18.1)	14.7 (12.4 to 16.9)
Placebo	118	19.8 (18.6 to 21.0)	13.9 (12.2 to 15.5)	11.9 (10.1 to 13.7)
Difference†		0.5 (−1.4 to 2.4)	2.4 (−0.0 to 4.8)	2.8 (0.5 to 5.1)
CReSS:				
Lansoprazole	102	50.3 (44.9 to 55.7)	38.9 (33.4 to 44.3)	36.6 (29.8 to 43.5)
Placebo	118	51.1 (46.4 to 55.8)	34.7 (29.6 to 39.9)	31.8 (26.6 to 36.9)
Difference†		−0.8 (−7.9 to 6.3)	4.2 (−3.2 to 11.6)	4.8 (−3.5 to 13.1)
LPR-HRQL:				
Lansoprazole	102	28.9 (24.5 to 33.3)	20.5 (16.1 to 25.0)	18.8 (13.7 to 23.8
Placebo	118	26.5 (22.5 to 30.5)	17.1 (13.3 to 21.0)	13.9 (10.0 to 17.8)
Difference†		2.4 (−3.5 to 8.3)	3.4 (−2.4 to 9.2)	4.9 (−1.3 to 11.1)

RSI=reflux symptom index; RSI-HB=laryngopharyngeal RSI items without the heartburn score; CReSS=comprehensive reflux symptom score; LPR-HRQL=laryngopharyngeal health related quality of life.

* Primary outcome measure.

† Lansoprazole minus placebo is the difference in means (95% confidence intervals).

**Table 3:** Multilevel mixed effect linear regression models for primary outcome of reflux symptom index score at 16 weeks

	β* (95% CI)	SE (β)	Test statistic	P value
Compliant ITT group (n=220)†:				
Lansoprazole (ref placebo)	1.93 (−0.35 to 4.20)	1.16	1.66	0.096
RSI-HB baseline severity severe (ref mild)	8.17 (5.86 to 10.49)	1.18	6.92	<0.001
Constant	14.35 (8.38 to 20.32)	3.04	4.71	<0.001
Pragmatic ITT group (n=267)†:				
Lansoprazole (ref placebo)	1.47 (−0.60 to 3.53)	1.06	1.39	0.17
RSI-HB baseline severity severe (ref mild)	7.44 (5.35 to 9.54)	1.07	6.95	<0.001
Constant	15.17 (9.27 to21.08)	3.01	5.03	<0.001

ITT=intention to treat; RSI-HB=laryngopharyngeal RSI items without the heartburn score.

* Adjusted by site (random effect).

† Estimate of treatment effect defined as estimated difference in 16 week score between randomised arms after adjustment for site and baseline severity.

After adjustment for other important clinical and personal baseline factors, and when analysing reflux finding score as a continuous measure, secondary analyses of the primary outcome in the wider pragmatic intention-to-treat group (table 3) gave similar results. No statistically significant difference was found in the RSI score at 16 weeks between the treatment groups in any of these planned analyses. At 12 months, RSI scores in participants in the lansoprazole group were 2.5 points higher (worse) than those in participants in the placebo group (95% confidence interval −0.1 to 5.0, P=0.06) (see supplementary table 5).

### Secondary outcome measures

Analysis of the RSI-HB showed that the mean RSI score at 16 weeks in the lansoprazole group was 2.4 points higher (worse) than in the placebo group: 16.3 (95% confidence interval 14.5 to 18.1) *v* 13.9 (12.2 to 15.5) (table 2).

The CReSS scores improved (reduced) from baseline to 16 weeks in both treatment groups (table 2 and supplementary table 6).

The mean laryngopharyngeal health related quality of life scores showed similar noticeable improvement at 16 weeks in both treatment groups (table 2 and supplementary figure 1). In multilevel modelling the estimated overall laryngopharyngeal health related quality of life outcome score in the lansoprazole group was on average 2.9 higher (worse) than that in the placebo group (95% confidence interval −4.3 to 10.1; P=0.43).

Reflux finding scores at baseline were available for 256 participants (80% in the lansoprazole arm and 72% in the placebo arm). Mean baseline reflux finding scores were 9.7 (SD 4.1) in the lansoprazole group and 9.2 (3.8) in the placebo group. The baseline scores were not significantly related to the RSI score at 16 weeks using first order fractional polynomial transformations.

### Discussion

This study found that lansoprazole offers no benefit over placebo for patients with persistent throat symptoms. No trends were in favour of lansoprazole. Patients who received lansoprazole on average reported worse improvement in symptoms than those receiving placebo. Treating patients for reported persistent throat symptoms “empirically” with PPIs, in the absence of specialist investigations, is common practice by healthcare practitioners worldwide. This should now be discouraged through evidence based treatment guidelines. Recent guidelines on chronic cough, which previously advocated trials of PPIs for presumed reflux related symptoms, have incorporated high level evidence and placebo controlled trials of PPIs and now state that acid reduction treatments should not be routinely prescribed for this condition.^24^

The practice of prescribing PPIs for these patients is based on several observational cases series showing improvement in symptoms over time with treatment. The inability of placebo controlled trials to replicate the benefits of PPIs in uncontrolled observational studies, however, suggests a misattribution of placebo enhanced spontaneous resolution in such single cohort reports.^15^ ^18^ A systematic review of studies that used PPIs as empirical treatment for suspected reflux related throat symptoms identified 14 uncontrolled studies, one non-blind, non-randomised study with a control group of healthy volunteers, and six double blind, placebo controlled randomised trials from 1994 to 2004.^7^ A lack of common outcome measures, potential selection bias, or inadequate blinding of the results were among typical limitations. An updated meta-analysis to 2005 of eight randomised controlled trials concluded that PPI treatment “may offer a modest but non-significant clinical benefit” over placebo.^25^ A previous trial randomised 145 patients in a 2:1 ratio to esomeprazole twice daily or matched placebo twice daily.^8^ The participants completed a Likert scale assessment of five symptoms: throat clearing, cough, globus, sore throat, and hoarseness. The participants identified their single most bothersome symptom at baseline. The primary outcome measure was the percentage of participants who had resolution of their most troublesome symptom. No difference was found between the treatment groups for the primary outcome measure, nor in secondary outcomes of laryngeal appearances, pH monitoring, or disease specific quality of life (laryngopharyngeal health related quality of life). Although discussions about methodology could be raised, it is difficult to conclude why this study did not diminish the enthusiasm for PPI use in this patient population. In our multicentre trial, we used a validated symptom reporting outcome. It is imperative that high quality clinical trials with negative outcomes, such as our trial, are incorporated into evidence based guidelines to bring about practice change. Our trial provides evidence for the medical profession to question indiscriminate use of PPIs and change empirical practices.

### Strengths and limitations of this study

The strengths of our trial are that it was performed in several centres, reflecting national practices; comprised a representative patient population; was fully powered; and minimised bias through blinding.

Drop-out rate and compliance are problems in pragmatic clinical trials with patient reported outcome measures. Our trial was designed to recruit 266 patients with complete primary patient reported outcome data to detect a clinically important difference with 90% power. We recruited 346 patients, assuming a 20% drop-out rate, and a total of 267 patients completed the primary outcome measure (pragmatic set): 220 within the protocol timescale (compliant set). Drop-out was observed as anticipated, and the RSI questionnaires returned at 16 weeks were fully completed.

We recruited a realistic patient population providing generalisable results across NHS clinics. Our trial specifically assessed the effectiveness of lansoprazole for patients managed within a secondary care setting. However, the results seem essentially applicable to any proton pump inhibitor when used for the treatment of patients with persistent throat symptoms in primary and secondary care. No evidence was found to show superiority of one PPI over another for GORD, for which PPIs are well established as effective treatment. No such evidence exists for persistent throat symptoms either. The range of symptom severity in our trial included a few values that overlapped with the general population, based on the total RSI score. The range of symptom severity in participants in our trial thus reflects the spectrum of symptom severity encountered in primary care. It would be reasonable to assume that if lansoprazole, and by inference any PPI, is not effective for the population of patients recruited into our trial, then the drug would also be no more effective than placebo for patients with less severe symptoms.

One quarter of participants recruited to our trial had been prescribed a PPI in the preceding 12 months. The inclusion of these participants, after the appropriate wash-out period, was justified as it reflects a commonly encountered patient pathway within our pragmatic clinical trial design, and most receive a short course of a once daily regimen of PPI within primary care. This is a reasonable treatment trial for suspected heartburn or GORD, but our results show that patients whose throat symptoms respond to PPI are equally likely to respond to a placebo. Moreover, the PPI regimen for laryngopharyngeal symptoms in secondary care typically is twice daily for two to six months.^10^ Few if any of our participants who had previously tried PPIs had received this intensity of treatment, not least because doctors typically refer early to exclude occult disease.

Our trial could be criticised for lacking any objective measure of GORD within the methodology or for employing any such test as an inclusion criteria. However, we did address the use of PPIs in an empirical setting, which was a near universal practice at the time of our study. The use of techniques such as pH testing with impedance manometry is not common within otolaryngology practices in the UK. The inclusion of this technique, or of others, would have led to far greater expense to the trial funder, reduced the recruitment rate, and narrowed the trial’s applicability to specialist practice only. We recognise that many patients presenting with persistent throat symptoms have coexisting symptoms of GORD, as GORD symptoms are present in up to 20% of the population, and that these traditional symptoms of heartburn are commonly treated with PPIs. When the RSI was assessed through a postal survey in 378 respondents, about 50% of patients who met the criteria suggesting that persistent throat symptoms were due to reflux did so on the basis of their high traditional GORD symptom scores.^16^ We assessed the effectiveness of PPIs on persistent throat symptoms alone and not on coexisting symptoms of classic GORD. In adopting the inclusion criterion and a baseline severity stratification that removed the polysymptomatic GORD item from the RSI, we ensured that the outcomes pertained to persistent throat symptoms alone. The question remains as to how coexisting throat and GORD symptoms should be managed, and it is for this group of patients that research into specialist oesophageal investigations could be focused. When assessing the individual items of the RSI (see supplementary file), item 9, covering traditional GORD symptoms, generally had low reported scores and did not over 16 weeks change appreciably more in one group than the other. This observation suggests that it would be inappropriate to perform subgroup analysis of patients with higher GORD symptoms at baseline.

### Unanswered questions and future research

Exploring alternative strategies to manage persistent throat symptoms requires well designed clinical trials, but these will only be possible when the practice of prescribing PPIs for these symptoms is discouraged. In our trial we found symptoms improved equally over time between PPIs and placebo, but patients’ symptoms did not reduce to those of the general population. Hence a clear need exists to investigate more effective treatment strategies. Our results might support the renewed focus of research into the well established psychological concomitant throat symptoms in some patients—namely, anxiety, distress, depression, and coexisting persistent physical symptoms.^26^ ^27^ ^28^ Strategies that employ the techniques of reattribution (offering alternative explanations for causes of the symptoms), adjustments to lifestyle, and behaviour modification of speech or cognitive behavioural therapy^28^ ^29^ ^30^ ^31^ ^32^ seem to be relevant and a reasonable focus of further research. For such a common condition as persistent throat symptoms, it would seem appropriate to investigate whether elements of specialist proven treatments such as cough suppression techniques, voice therapy, management of globus, and cognitive behavioural therapy could be adapted into a clinically and cost effective self-directed care package for patients.

Great clinical interest has been shown in attributing throat and upper airway symptoms to manifestations of GORD. Interest is growing in weakly acidic, or non-acidic, reflux, which would intuitively seem less likely to respond to PPIs yet contains the other important elements of gastric contents. Little evidence exists for the role of other factors that might reduce reflux related persistent throat symptoms, such as diet,^33^ lifestyle,^34^ and alginates.^23^ Our trial does not refute reflux as a cause or contributing factor for some patients’ symptoms, and although reflux of gastric contents containing pepsin might be implicated in some patients, defining such individuals and appropriate management needs further research. This requires clarification on the use and interpretation of specialist investigations to identify reflux episodes and response to treatments.

### Policy implications

No evidence supports the empirical use of PPIs to treat persistent throat and voice symptoms. The lack of any trend towards benefit with lansoprazole in our trial should discourage subgroup analysis hypotheses. The trial’s conclusions are particularly apt for the non-specialist to whom the message has filtered through from otolaryngology case series evidence that PPIs are appropriate for this patient population. Our results also might be explained by an underestimation of the placebo effect in this group of patients and the failure of PPIs to affect non-acidic, or weakly acidic, reflux episodes.

### Conclusions

A regimen of lansoprazole twice daily offered no symptomatic benefit over matched placebo for patients with persistent throat symptoms. Evidence supporting the empirical use of PPIs to treat persistent throat symptoms is lacking.

**What is already known on this topic**

Throat symptoms are a common reason for referral from primary to secondary care—the sensation of a lump in the throat affects up to half of the general population at some stage
Proton pump inhibitors (PPIs) are widely used in both primary and secondary care in the UK as empirical treatment for throat symptoms
Published meta-analyses of PPIs for the treatment of throat symptoms include small scale studies of limited value

**What this study adds**

This trial found no evidence of benefit for patients with persistent throat symptoms who were treated empirically with a PPI

## Web extra

Extra material supplied by authors

## Notes

Contributors: JO’H and DDS are joint first authors. JO’H, PC, and JAW conceived and designed the study. All authors acquired, analysed, or interpreted the data. DDS and TF performed the statistical analysis. JO’H, DDS, and JAW drafted the manuscript. All authors critically revised the manuscript for important intellectual content. JO’H and JAW are the guarantors. JO’H is the corresponding author and attests that all listed authors meet authorship criteria and that no others meeting the criteria have been omitted.

Ethical approval: This study was approved by the National Research Ethics Service Committee-North East: Tyne and Wear South (reference No 13/NE/0336).

Data sharing: The authors are committed to making the relevant anonymised patient level data available on request, in line with Newcastle University’s research data management guidance.

The lead author and manuscript guarantor (JO’H) affirms that the manuscript is an honest, accurate and transparent account of the study being reported; that no important aspects of the study have been omitted; and that any discrepancies from the study as originally planned and registered have been explained.

Dissemination to participants and related public communities; Recruited patients have been informed that the results of the trial will not be sent to individuals but will be available after publication on the trial website (www.toppits.co.uk).

Provenance and peer review: Not commissioned; externally peer reviewed.
