# Supplementary material for: Use of proton pump inhibitors to treat persistent throat symptoms: multicentre, double blind, randomised, placebo controlled trial
Source: BMJ. 2021 Jan 7;372:m4903. doi: 10.1136/bmj.m4903 (PMC7789994; doi:10.1136/bmj.m4903)

The Trial of Proton Pump Inhibitors in Throat Symptoms – Supplementary Appendix

Contents:

Table 1 The Reflux Symptom Index 2
Table 2 Baseline demographic characteristics and stratification variables, all randomised patients and pragmatic ITT population 3
Table 3 Individual RSI item median scores at baseline and 16 weeks, for the compliant population.... 6
Table 4 Primary outcome measure RSI, pragmatic ITT population.. 8
Table 5 Multilevel mixed effect linear regression at 12 months, compliant ITT population................. 8
Table 6 Comprehensive Reflux Symptom Score total scores at 16 weeks, compliant population......... 9

Figure 1 Reflux Symptom Index score at baseline and follow-up visits for the compliant population (n=220) (median, interquartile range (IQR), upper and lower adjacent values (+/-1.5xIQR) and outlier) 5
Figure 2 Laryngopharyngeal Reflux Health Related Quality of Life, compliant population. 9

Table 1 The Reflux Symptom Index

Permission to use in the trial and reproduce for publication obtained from the authors. The use of the Reflux Symptom Index (RSI) without the 9th item (RSI- Heartburn score, RSI-HB) was devised specifically for TOPPITS.

Within the last MONTH, how did the following problems affect you?

0 = no problem 5 = severe problem

Hoarseness or a problem with your voice	0	1	2	3	4	5
Clearing your throat	0	1	2	3	4	5
Excess throat mucus or postnasal drip	0	1	2	3	4	5
Difficulty swallowing food, liquids or tablets	0	1	2	3	4	5
Coughing after eating or lying down	0	1	2	3	4	5
Breathing difficulties or choking episodes	0	1	2	3	4	5
Troublesome or annoying cough	0	1	2	3	4	5
Sensation of something caught in your throat or a lump in your throat	0	1	2	3	4	5
Heartburn, indigestion or stomach acid coming up	0	1	2	3	4	5

**TOTAL**		**TOTAL minus heartburn score**	

Table 2 Baseline demographic characteristics and stratification variables, all randomised patients and pragmatic ITT population

		All randomised patients (n=346)			Pragmatic ITT population (n=267)		
Variable		Lansoprazole	Placebo	Total	Lansoprazole	Placebo	Total
Gender	Male	71 (41%)	79 (45%)	150 (43%)	49 (39%)	65 (46%)	114 (43%)
	Female	101 (59%)	95 (55%)	196 (57%)	78 (61%)	75 (54%)	153 (57%)
Age (years)	Mean (SD)	53.5 (13.3)	50.8 (13.9)	52.2 (13.7)	54.8 (12.8)	52.3 (13.7)	53.5 (13.3)
	Range	(21,84)	(20,80)	(20, 84)	(23, 84)	(21, 80)	(21,84)
Body Mass Index	n	169	170	339	125	140	265
	Mean (SD)	28.2 (5.9)	28.1 (5.3)	28.1 (5.6)	28.1 (6.3)	28.1 (5.3)	28.1 (5.8)
	Range	(11.3,56.9)	(18.3,49.1)	(11.3,56.9)	(11.3,56.9)	(18.3,49.1)	(11.3,56.9)
RSI	n	171	171	342	127	140	267
	Mean (SD)	21.7 (7.4)	22.1 (7.0)	21,9 (7.2)	21.7 (7.4)	21.9 (7.0)	21.8 (7.2)
	Range	(10,41)	(10,43)	(10,43)	(10, 41)	(10, 43)	(10, 43)
RSI-HB	n	171	171	342	127	140	267
	Mean (SD)	20.0 (6.8)	20.1 (6.5)	20.1 (6.6)	20.0 (6.9)	20.0 (6.5)	20.0 (6.7)
	Range	(10,38)	(10,38)	(10,38)	(10,38)	(10,38)	(10,38)
Severity category*	Mild	91 (53%)	93 (53%)	184 (53%)	69 (54%)	72 (51%)	141 (53%)
	Severe	81 (47%)	81 (47%)	162 (47%)	58 (46%)	68 (49%)	126 (47%)

* Stratification Factor at Randomisation: Mild (10≤RSI-HB≤20), Severe (RSI-HB>20)

Patient Reported Satisfaction

At the 12-month follow-up (visit 3), 213 out of 346 (62%) participants answered the satisfaction question, of whom 115 (54%) were very satisfied, 59 (28%) were satisfied, 29 (14%) were neither satisfied nor dissatisfied, five (2%) were dissatisfied and five (2%) were very dissatisfied.

Figure 1 Reflux Symptom Index score at baseline and follow-up visits for the compliant population (n=220) (median, interquartile range (IQR), upper and lower adjacent values (+/-1.5xIQR) and outlier)

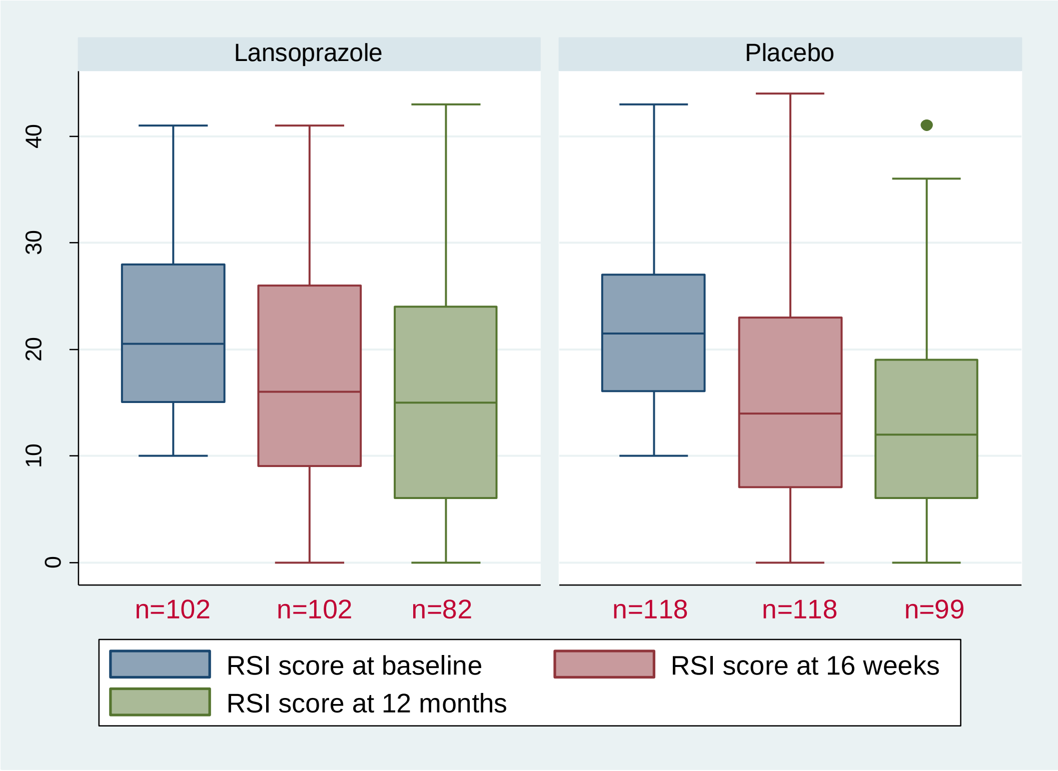

Table 3 Individual RSI item median scores at baseline and 16 weeks, for the compliant population.

RSI items	Lansoprazole group median score (LQ, UQ) n=102			Placebo group median score (LQ, UQ) n=118		
	baseline	16 week	change	baseline	16 week	change
1 - Hoarseness	2.5 (1,4)	1 (0, 3)	0 (-1, 0)	3 (1, 4)	2 (0, 3)	0 (-2, 0)
2 – Throat clearing	4 (3, 4)	3 (1, 4)	0 (-2, 0)	3 (3, 4)	2 (1, 4)	-1 (-2, 0)
3 - Excess throat mucus or postnasal drip	3 (2, 4)	3 (1, 4)	0 (-1, 0)	3 (2, 4)	2 (1, 4)	-1 (-2, 0)
4 - Difficulty swallowing food, liquids or tablets	1 (0, 3)	1 (0, 2)	0 (-1, 0)	1 (0, 3)	0 (0, 2)	0 (-2, 0)
5 - Coughing after eating or lying down	2 (0, 3)	1.5 (0, 3)	0 (-1, 0)	2 (1, 4)	1 (0, 3)	0 (-2, 0)
6 - Breathing difficulties or choking episodes	1 (0, 3)	0 (0, 3)	0 (-1, 0)	1 (0, 3)	0 (0, 2)	0 (-1, 0)
7 - Troublesome or annoying cough	3 (1, 4)	2 (0, 4)	0 (-1, 0)	3 (1, 4)	1 (0, 3)	-0.5 (-2, 0)
8 - Lump in the throat sensation	4 (3, 5)	3 (1, 4)	-1 (-2, 0)	4 (2, 5)	2 (1, 4)	-1 (-2, 0)

9 - Heartburn, indigestion or stomach acid coming up	1 (0, 3)	1 (0, 2)	0 (-1, 0)	2 (1, 3)	1 (0, 3)	0 (-1, 1)

LQ = Lower Quartile, UQ = Upper Quartile

Table 4 Primary outcome measure RSI, pragmatic ITT population

	**Baseline (Visit 1)**		**16 weeks (Visit 2)**	
	Lansoprazole (N=127)	Placebo (N=140)	Lansoprazole (N=127)	Placebo (N=140)
Median (IQR)	21 (16, 26)	22 (16, 27)	15 (9, 25)	15 (8, 23)
Mean (SD); CI	21.7 (7.4)	21.9 (7.0)	17.1 (9.6); 15.5, 18.8	16.0 (9.5); 14.4, 17.6
Range	(10, 41)	(10, 43)	(0, 41)	(0, 44)

CI = 95% confidence internal about mean

Table 5 Multilevel mixed effect linear regression at 12 months, compliant ITT population

Model (n=185)*	β	se (β)	Test statistic	p-value	95% CI (β)	
					lower	upper
Group (ref = Placebo) Lansoprazole	2.469	1.311	1.88	0.060	-0.100	5.038
RSI-HB baseline severity: (ref =Mild) Severe	8.233	1.314	6.27	<0.001	5.658	10.808
Constant	19.149	4.405	4.35	<0.001	10.515	27.784

Table 6 Comprehensive Reflux Symptom Score total scores at 16 weeks, compliant population.

CReSS	Total (range: 0-170)	
	Lansoprazole	Placebo
n	102	118
Median (LQ, UQ)	36 (15,55)	27.5 (14,48)
Mean (SD)	38.9 (27.7)	34.7 (28.3)
95% CI	(33.4, 44.3)	(29.6, 39.9)
Range (min, max)	(2,140)	(0,158)

Figure 2 Laryngopharyngeal Reflux Health Related Quality of Life, compliant population.

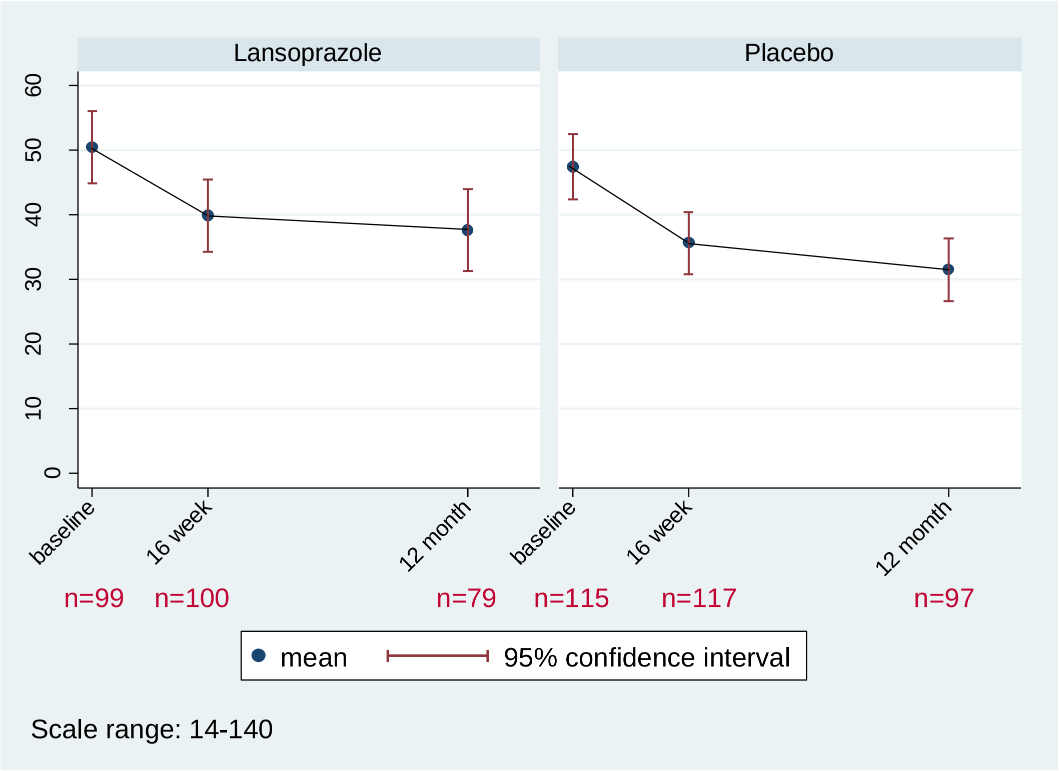

Supplement: Supplementary file 1 — Supplementary information: additional tables 1-6 and figures 1 and 2 [file ohaj058384.ww.pdf]
